# Early results of excision of 220 cases of primary chest wall tumors in 12 year period

**DOI:** 10.1186/1749-8090-10-S1-A16

**Published:** 2015-12-16

**Authors:** Amer Bilal

**Affiliations:** 1 Dept of Cardiothoracic Surgery, Lady Reading Hospital, Peshawar, Pakistan

## Background/Introduction

Primary chest wall tumors originate from different constructions of thoracic wall. Resectable tumors are excised with primary closure or chest wall reconstruction with better outcome.

## Aims/Objectives

To assess the surgical outcomes in primary chest wall tumor.

## Method

220 patients from June 2002 to Dec 2014 were retrospectively analyzed. Patients of all ages, both sexes and operable primary chest wall tumor were included. Clinical evaluation, routine investigations, chest radiographs, computed tomography and biopsy were done. Incisional biopsy was done for >5 cm mass while excisional biopsy was done in smaller tumors. Complete excision of the chest wall tumor with 5 cm free margin and one normal rib above and one normal rib below was done. Specimen was sent for histopathology. In skeletal reconstruction plastic surgeon was involved. Patients sent to oncologist for adjuvant therapy accordingly. One year follow-up were done.

## Results

Out of 220 patients, 143 were male and 77 were female, age ranges from 9-80 years with a median of 27.8 years. 151 patients experienced painless mass and 69 patients painful mass.113 chest wall masses presented on right side, 70 left sided and 37on sternum. Sizes were <3 cm 78, 3-5 cm 92, 5-10 cm 42 and >10 cm 08. Chest wall resection and primary closure was done in 107 cases while in 113 cases resection and reconstruction done using marlex mesh alone in 98 cases and reinforced with methyl methacrylate in 15cases. Histologically Chondrosarcoma was reported in 61.5%, Fibrosarcoma in 25%, Ewing sarcoma in 11.5% while 2% specimens were reported as chondroma. Postoperative flail observed in 8cases, 5 patients died despite prolonged ventilation. All patients referred to oncologist post operatively. One year follow up of all 215 alive patients were tumor free.

## Discussion/Conclusion

Primary chest wall tumor can be safely managed by resection and primary closure or chest wall reconstruction and are associated with long term survival.

